# Current Trends in In Vitro Diagnostics Using Surface-Enhanced Raman Scattering in Translational Biomedical Research

**DOI:** 10.3390/bios15050265

**Published:** 2025-04-22

**Authors:** Sitansu Sekhar Nanda, Dae-Gyeom Park, Dong Kee Yi

**Affiliations:** 1 Department of Chemistry, Myongji University, Yongin 17058, Republic of Korea; nandasitansusekhar@gmail.com; 2 Advanced Refrigeration and Air-Conditioning Energy Center, Pusan National University, Busan 46241, Republic of Korea

**Keywords:** mRNA, Raman spectroscopy, biomarker, SERS, AI

## Abstract

Immunoassays using surface-enhanced Raman scattering (SERS) are prosperous in disease diagnosis due to their excellent multiplexing ability, high sensitivity, and large dynamic range. Given the recent advancements in SERS immunoassays, this work provides a comprehensive overview, from fundamental principles to practical applications. An mRNA sensor utilizing Raman spectroscopy is a detection method that leverages the unique vibrational characteristics of mRNA molecules to identify and quantify their presence in a sample, often achieved through a technique called SERS, where specially designed nanoparticles amplify the Raman signal, allowing for the highly sensitive detection of even small amounts of mRNA. This review analyzes SERS assays used to detect RNA biomarkers, which show promise in cancer diagnostics and are being actively studied clinically. To selectively detect a specific mRNA sequence, a probe molecule (e.g., a DNA oligonucleotide complementary to the target mRNA) is attached to the SERS substrate, allowing the target mRNA to hybridize and generate a detectable Raman signal upon binding. Thus, the discussion includes proposals to enhance SERS immunoassay performance, along with future challenges and perspectives, offering concise, valid guidelines for platform selection based on application.

## 1. Introduction

Creating fast, highly sensitive tests for disease markers in blood has been a major goal in medical diagnostics, prompting lots of research. Clinical trials often utilize immunoassay methods like electrochemiluminescence (ECL) and Enzyme-Linked Immunosorbent Assay (ELISA) to measure specific protein biomarkers quantitatively [1]. Many newly found disease biomarkers exist at very low concentrations (below ng/mL), and thus conventional ECL or ELISA methods cannot detect them. ELISA, for example, uses enzyme-mediated color change to detect biomarkers; however, this change is undetectable by a microplate reader at low concentrations [2]. Likewise, clinical labs frequently use ECL for luminescence detection, but it faces challenges with biomarkers having very low cut-off values. Recently, the electromagnetic enhancement properties of nanostructures used in SERS technology have shown promise in overcoming these limitations [3]. This review examines current translational biomedical research using SERS-based in vitro diagnostic techniques.

**Figure 1:**
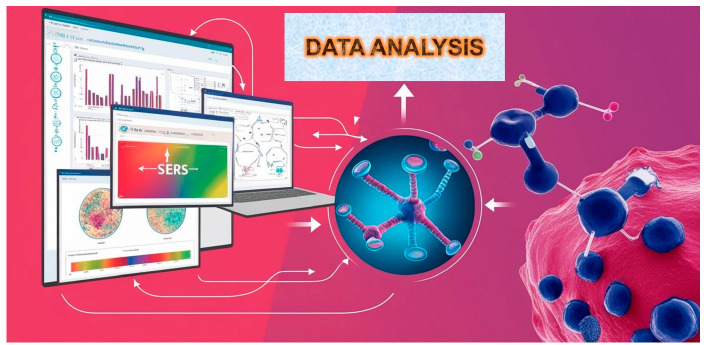
Machine learning can distinguish healthy from diseased states by analyzing the unique Raman spectra generated by the powerful spectroscopic technique, SERS, based on molecular composition.

SERS, a two-photon inelastic scattering technique, is a powerful bioanalytical tool; this is especially true when combined with plasmonic nanogap structures. This combination helps SERS overcome sensitivity problems in ECL and fluorescence detection, which are often limited by quenching [4,5,6,7]. When light hits free electrons on gold or silver nanostructures, the electromagnetic field gets much stronger. This phenomenon goes by the name of localized surface plasmon resonance (LSPR). When target molecules are near nanostructures, especially in tiny gaps (under 10 nm), the LSPR effect boosts their Raman scattering signal intensity significantly. This improved signal allows SERS to surpass the sensitivity limits of traditional methods like ECL and fluorescence, commonly used in medical diagnostics. The use of SERS bioassays is presented in Figure 1, highlighting the role of integrated microdevices, portable Raman readers, and artificial intelligence (AI).

However, the transition of SERS technology from lab research to clinical use faces significant hurdles requiring attention. SERS offers a key advantage: amplifying Raman signals at specific hotspots while cutting background noise, thus boosting the signal-to-noise ratio. Although highly sensitive, Raman signal enhancement relies heavily on precise nanogap control for electromagnetic enhancement. Therefore, creating dependable SERS substrates with uniform, controlled nanogaps presents a significant hurdle [8,9]. This is particularly crucial for SERS-based POCT in vitro diagnostics, demanding highly reproducible measurements. Achieving reproducibility gets even harder when combining SERS detection with microdevices like LFAs or microfluidic channels; this is critical.

Historically, SERS’s use has been limited to labs because of reproducibility problems and the size of Raman spectrometers, hindering its widespread use as a diagnostic tool. But lately, portable SERS systems are being revolutionized by advancements in miniaturized optical devices and nanotechnology. Advanced nanotechnologies enable the creation of plasmonic substrates with consistently uniform nanostructures, thus boosting Raman signal reproducibility. Portable Raman spectrometers and technological advancements have significantly boosted SERS’s clinical diagnostic capabilities. Replacing traditional ECL or fluorescence labels with Au or Ag nanoparticles that create hotspots allows for highly sensitive biomarker detection—surface-enhanced Raman scattering (SERS) offers single-molecule sensitivity, achieving enhancement factors [10,11,12]. Therefore, multiple studies have investigated SERS technology’s use in diagnosing cancer or COVID-19 biomarkers in vitro [13,14,15].

The need for novel, highly sensitive optical measurement methods for biomarker detection is growing, as evidenced by the detection of biomarkers in human biofluids at extremely low concentrations. This review examines the newest progress in SERS-based in vitro diagnostics, a potentially highly sensitive method. Lastly, we covered the key factors needed for the successful clinical translation of SERS-based detection from the lab.

## 2. History of Developing Immunoassays Based on SERS

Among the many immunoassay detection methods, surface-enhanced Raman spectroscopy (SERS) has become increasingly popular lately. This review centers on the progress of SERS-based immunoassays. SERS-based immunoassays utilize surface-enhanced Raman scattering to generate readout signals. Raman scattering, the inelastic scattering of photons, is famously known to have been discovered by Raman and Krishnan in liquids [16] and by Landsberg and Mandelstam in crystals [17]. Raman scattering offers detailed structural and quantitative insights into virtually any molecule or material. However, Raman spectroscopy’s weak signals restrict its use in biomedical and chemical research. Fleischmann et al. found in 1973 that pyridine molecules on a rough silver surface showed a greatly amplified Raman scattering signal [18]. With enhancement factors ranging from 10^3^ to 10^10^ [19,20,21], this method has seen widespread use in ultrasensitive biochemical analysis.

A “sandwich” scheme was used to develop the first common SERS immunoassay in 1999 [22]. In that protocol, capture antibodies are bound to a flat gold surface to form an immune substrate. Raman reporters labeled gold nanoparticles were conjugated with detection antibodies, creating SERS probes. Subsequently, antibodies on the substrate could capture the target antigens, which the SERS probes would then recognize. The characteristic SERS spectrum of Raman reporters reveals the presence and concentration of a specific antigen. Importantly, the authors showed that their method also worked for multiplex detection using SERS encoding.

Over the next few decades, SERS-based immunoassay platforms have rapidly evolved from solid (metallic [22] and nonmetallic [23]) and liquid (magnetic [24] and nonmagnetic [25]) phases to microfluidic chips [26], paper devices [27], and optical fibers [28]. The wide variety of SERS-active platforms provides ample options for analyzing proteins, disease biomarkers, ions, toxins, bacteria, viruses, cells, and more. Conversely, a high-throughput, multiplex analysis of numerous analytes within single samples is increasingly necessary, particularly in clinical diagnostics, drug discovery, and environmental monitoring [29]. Significantly, the SERS-based optical-encoding technique enables the high-throughput detection of multiple targets simultaneously, distinguished by Raman frequency or intensity. Additionally, SERS has been coupled with fluorescence [30] or spatial coordinates [31] for combined encoding. Combining SERS with other methods significantly boosted the encoding capacity, crucial for high-throughput analysis.

To summarize, SERS-based immunoassays offer three key advantages. Firstly, SERS detection boasts high sensitivity, reaching single-molecule levels thanks to its large enhancement factor (~10^10^), proving highly valuable for trace analysis [32,33,34]. Secondly, highly efficient spectroscopic encoding is facilitated by using narrow Raman bands, which enables high-throughput detection [35]. Thirdly, SERS is immune to photobleaching and quenching [36]. Repetitive signal measurements, enabled by the high stability of SERS signals, minimize testing errors. Because of these advantages, SERS immunoassays are used extensively in research and practical applications.

## 3. Biomedical Diagnostics

The global rise of new biological threats has spurred the development of faster, more efficient methods for the early detection and assessment of viral pathogens. Researchers are constantly working to create better, faster, and more accurate ways to detect dangerous pathogens early in disease monitoring. Viral detection is commonly used by PCR and ELISA techniques. However, these methods have some critical drawbacks: (i) they are time-consuming, (ii) they necessitate a complex initial isolation step for the target organism, (iii) they involve extensive incubation and purification processes, and (iv) they are restricted to highly specific antibodies or primers, hindering rapid on-site detection. The early diagnosis of viral infections is now made ultra-efficient, cheap, and rapid using recently developed SERS-based biosensors [37,38,39,40,41,42,43,44]. 

Different alloy and biomolecule-based SERS immunoassays were developed for Hepatitis B virus detection [41,45,46]. Scientists recently developed a highly sensitive SERS immunoassay on a microfluidic chip for Hepatitis B virus detection, using a novel Raman-reported active substrate [41]. A microfluidic chip with Hepatitis B surface antigen (HBsAg) antibodies identified Hepatitis in human blood plasma. Using Raman reporter basic fuchsin improved SERS effectively and bound well to Au nanostructures and antibodies. The sandwich structure of the antigen and antibody resulted from this fuchsin (FC)-labeled gold nanoflowers immunoassay. Using EDC/NHS coupling chemistry and a 6-amino-1-hexanethiol (AHT) layer, we attached the sandwich structure to an Au–Ag-coated gallium nitride (GaN) SERS substrate. The high stability, reproducibility, and strong surface enhancement of this SERS-active substrate proved critical to improving SERS immunoassay efficiency. Researchers used this immunoassay to detect Hepatitis B in human blood plasma. A calibration curve was generated by correlating the 1178 cm^−1^ SERS signal intensity to the antigen concentration for this purpose. Furthermore, HBsAg detection achieved high specificity with a calculated limit of detection of 0.01 IU/mL.

Respiratory tract infections (RTIs), such as bronchitis, pneumonia, and tracheitis, pose a significant global health problem. Accurately diagnosing RTIs is challenging due to the wide range of potential pathogens, such as bacteria, influenza A and B, parainfluenza viruses, and adenoviruses. Furthermore, delayed or incorrect RTI diagnoses might be fatal. Therefore, the precise and early detection of pathogens is crucial for patient treatment. These detection techniques, frequently used together, such as immunofluorescence assays (IFAs), can produce false positives when identifying antibodies in human serum. A lateral flow microarray with SERS nanotags was developed for the fast, sensitive detection of multiple RTI pathogens.

In ref. [47], influenza A, B; parainfluenza 1, 2, 3; and adenovirus detection limits were 0.031, 0.035, 0.030, 0.032, and 0.040 pM. Gold nanoparticles, functionalized with 4, 4′-thiobisbenzenethiol (TBBT) and PEG and conjugated with an influenza A antibody, were used to create SERS substrates [42,43,48]. A SERS immunoassay using 2D arrays of Au@Ag nanoparticles with an outer layer of Au/Ag significantly enhanced detection accuracy and sensitivity [49,50]. Antibody recognition of the selective nucleoprotein was found via TBBT SERS signal detection. Compared to flat gold substrates, these SERS substrates boosted detection power approximately fourfold. Employing an effective fabrication method for an Au@Ag 2D array, rather than a flat Au film, resulted in the enhanced sensitivity and efficiency of SERS-based biosensors. The influenza A virus was detected using this technique at a concentration of 6 TCID_50_/mL. Recently, researchers used SERS-based antibody probes for quick and easy influenza A virus detection [44]. This method simplified SERS-based antibody synthesis, avoiding complex reactions. This probe was made by simply mixing gold nanoparticles, Au-binding protein G, and an antibody. Utilizing SERS-based antibody probes, we achieved an assessment limit of 4.1 × 10^3^ TCID/mL, demonstrating the effective and selective detection of the influenza A virus (pH1N1).

**Figure 2:**
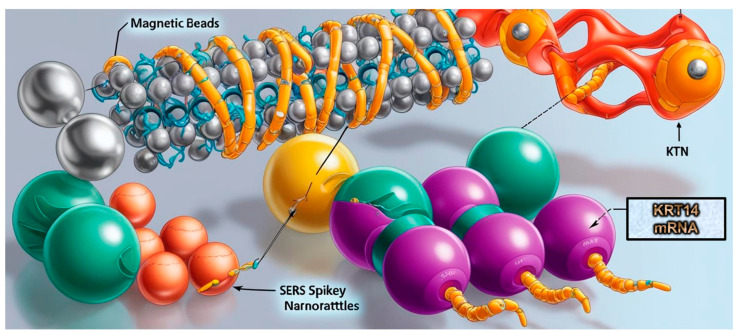
Spikey nanorattles (SpNRs), a new nanoparticle formulation, offer enhanced SERS due to their core-shell nanorattle architecture and a unique spikey outer coating. A DNA sandwich assay integrated SpNRs using magnetic-bead-functionalized capture probes and SpNR-functionalized reporter probes.

In areas lacking standard cancer diagnostics, early head and neck squamous cell carcinoma (HNSCC) detection is crucial for better patient outcomes. A sandwich hybridization approach employing magnetic beads and SERS spikey nanorattles (SpNRs) targets Keratin 14 (KRT14) mRNA, a potential HNSCC diagnostic biomarker (Figure 2). The assay accurately differentiated between HNSCC-positive and -negative tissues [51].

The COVID-19 pandemic has ravaged the globe over the past half-decade. COVID-19, or coronavirus disease 2019, is a strain of SARS-CoV-2 (severe acute respiratory syndrome coronavirus 2). The novel coronavirus was found to be a dangerous respiratory syndrome. COVID-19, a single-stranded RNA virus with a 30 kb genome, directly translates viral proteins via its mRNA. The SARS-coronavirus genome includes fourteen open reading frames (ORFs), with two-thirds of the 5′ end containing two ORFs. One-third of the 3′ genome encoded the four structural proteins: membrane, spike, envelope, and nucleocapsid. The spike protein, typically composed of two subunits (S1 and S2), plays a crucial role in attaching the virus to receptors on the host cell. The receptor-binding domain’s (RBD) crucial role in SARS-coronavirus binding makes it a potential therapeutic target for COVID-19 detection. For disease monitoring, early disease detection, ongoing disease tracking, and vaccine development, novel SERS-based methods were used [52,53,54,55,56].

Rapid, dependable, population-level COVID-19 testing is crucial for curbing community spread. Breath analysis for volatile organic molecules is promising, but current limitations include bulky equipment and inflexible analysis. A SERS breathalyzer uses changes in breath compound vibrational signatures, interacting with molecular receptors, to create a strong partial least squares discriminant analysis model for fast classification [57]. Utilizing nanotechnology, plasmonics, and 2D materials, Northern Arizona University’s Professor Miguel Jose Yacaman and his team developed a new non-invasive diagnostic method for rapid SARS-CoV-2 spike protein detection via single-molecule surface-enhanced Raman spectroscopy [58]. Israeli scientists also developed a rapid breath test using spectroscopy to detect coronavirus. A breath analyzer for detecting SARS-CoV-2 from exhaled volatile organic compounds was created using hybrid nanomaterial sensors. Controlled clinical studies in Wuhan (March 2020) [59] showed the breath analyzer detected COVID biomarkers in exhaled breath with 92% accuracy, 100% sensitivity, and 84% specificity.

Carlomango and his team also developed a Raman-spectroscopy-based detection technique for coronavirus using patient saliva samples. Raman spectroscopy analysis of saliva samples from COVID-19 patients yielded a unique biochemical signature, facilitating the development of a highly accurate, precise, specific, and sensitive diagnostic technique [60]. Sanchez et al. also used SERS to identify SARS-CoV-2 and its S and N proteins [61]. Robust SERS signals from ACE2 were detected at 1032, 1051, 1089, 1189, 1447, and 1527 cm^−1^ [62]. The ChAdOx1 vector derived from chimp adenovirus in the AstraZeneca vaccine confirmed the hypothesis that tears from AstraZeneca-vaccinated individuals resemble those of keratoconjunctivitis patients caused by adenovirus. Additionally, this approach consistently measured antibody signals, revealing up to three potential biomarkers for predicting vaccination status [63].

**Figure 3:**
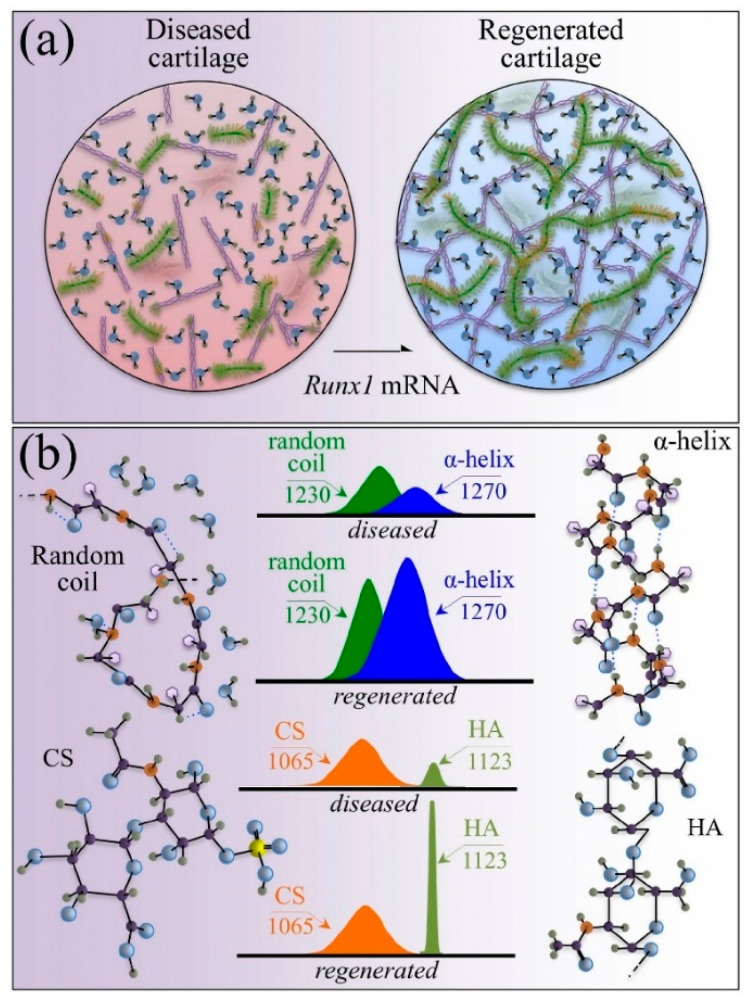
(**a**) Exploring the therapeutic role of Runx1 mRNA in activating chondrocytes. (**b**) Molecular-scale spectroscopic fingerprints of cartilage regeneration. This figure was adapted from [64] with permission.

Current Raman spectroscopy results consistently revealed several degradation processes in osteoarthritic cartilage. Runx1’s suppression of Osteoarthritis (OA) progression, achieved by primarily activating healthy chondrocytes, suggests that treatment will be most effective in the disease when more chondrocytes are available for mRNA delivery [64]. Raman spectroscopy validates the molecular-level therapeutic effects of Runx1 mRNA cartilage regeneration (Figure 3). Directly delivering mRNA into cells can solve safety and efficiency concerns, as demonstrated previously. Furthermore, this method prevented both inflammatory reactions (caused by mRNA’s poor immunogenicity) and mRNA degradation (from extracellular RNases). Raman spectroscopy will further clarify the therapeutic effects of the Runx1 mRNA procedure on cartilage regeneration. Ex vivo Raman spectra were obtained from chosen knee zones in mice before and after surgery and following mRNA treatment. The cartilage protein secondary structure (I_1240/I1270_), hydration (I_1312+1340_/I_1270_), glycosaminoglycan fraction variations (I_1063_/I_1126_), and N-glycan glycosylation (I_1165/I850_) were targets for analysis. Runx1 mRNA administration triggered molecular repair of the pathological abnormality, as evidenced by spectral shifts in Amide III and glycosaminoglycan ratios. Raman instruments’ compatibility with clinical arthroscope fiber optics means their proposed parameters could one day monitor OA in vivo and assess cartilage regeneration in real time at the molecular level.

Microbial Raman spectral outputs are demonstrably impacted by the growth stage [65,66,67,68,69,70,71]. For instance, nucleic acids’ Raman bands are stronger during exponential growth than in the stationary phase, indicating active DNA/RNA production in the exponential phase [65,66]. Raman spectral pattern changes caused by growth phases, plus cell-to-cell differences, might be mistaken for phenotype differences (like between bacterial species). Multivariate statistical methods, including PCA and discriminant analysis, have been used in numerous studies to identify bacterial species across different growth conditions [65,66,67,68,71]. However, a systematic investigation into how different growth phases affect the accuracy of AI-based bacterial discrimination using single-cell Raman data is lacking.

## 4. Portable SERS-Based Diagnostic Systems

**Figure 4:**
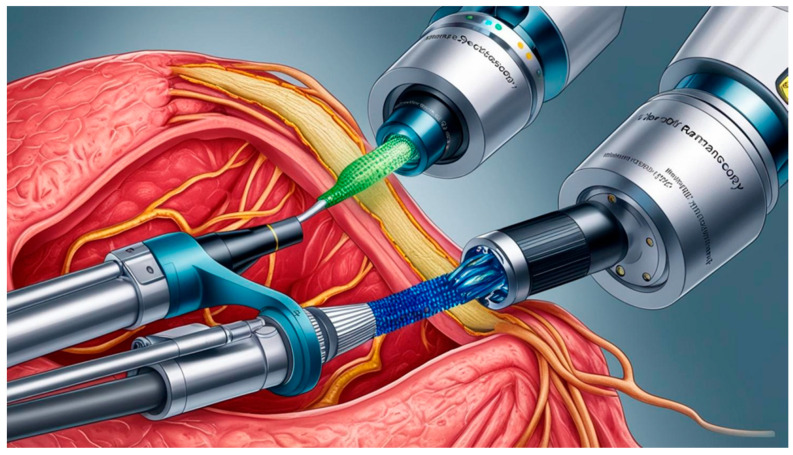
Raman endoscope applications in patient care. A Raman endoscope is inserted into the instrument channel of a standard clinical endoscope. A clinical endoscope’s Raman endoscope component is shown in this picture illuminating a spot inside a patient, as seen through the endoscope.

Benchtop Raman systems are designed for laboratory use, integrating advanced optical interfaces for sample manipulation and/or imaging, high-performance detectors, and multiple excitation wavelengths to enhance research flexibility. Complex software lets them automatically acquire and process signals. Because of their size, weight, and cost, they are unsuitable for use in field research. Portable, or “compact”, Raman spectrometers let users create streamlined, application-specific Raman systems while minimizing weight, size, cost, and complexity. If designed with an integrated excitation laser, they are compact, sturdy, and easily transported. These are also strong enough for use in many studies. Choices for excitation wavelength, integration level, and sample interface are offered. Data collection and analysis software is included; use a laptop or tablet to operate it. It is great for field research, taking measurements, and other uses. Early diagnosis and treatment improve patient survival rates and increase the likelihood of a cure. Currently, gold standard biopsies are effective for the prompt diagnosis of CTCs via blood samples. High-throughput screening in advanced labs is standard for most clinical and immunochemical tests. Using commercially available, portable devices and standard technologies offers increased control and analysis of samples. Portable PoC devices minimize reliance on large labs, enabling prompt, precise molecular biomarker detection. On-site evaluations and testing were enabled by advancements in PoC technology. Recently, scientists created PoC assays using SERS to directly detect cancer. Several cancer diagnostics, including tissue biopsies, could potentially use these assays. As an example, a SERS-based assay using micro-RNA biomarkers and neck cancer as a model was tested on 20 clinical samples, showing good accuracy in identifying squamous cell carcinoma. A portable Raman device, boasting 97% specificity, shows promise for PoC cancer diagnosis in under-resourced areas [72]. Endoscopic imaging and a special fiber optic Raman spectroscopy device are used in this minimally invasive method (Figure 4). Functioning as a clinical endoscope, this portable device uses SERS tags to identify and measure tumors, delivering multiplexed information in real time. An LOD of 326-fM (the concentration of SERS tags) with a working distance from 1 to 10 mm was achieved [73]. Additionally, it enables endoscopists to quickly differentiate between healthy and cancerous tissues. Rapid, on-site cancer diagnosis is enabled by using a highly sensitive SERS molecular imaging contrast agent. This contrast agent excels at recognizing and multiplexing, enabling the real-time detection of several cancer biomarkers. This technology offers real-time diagnostic data, facilitating quick and precise cancer diagnoses. A sophisticated Raman endoscope can also be used to identify spectral markers associated with targeted SERS tags. Shu et al. created a compact, portable system using SERS to properly screen and validate early-stage cancer prognoses [74].

## 5. In Vitro Transcription (IVT) Process Monitoring

Jaekle et al. [75] reported a new way to use Raman spectroscopy in cell line development, combining Raman microspectroscopy, laser-induced forward transfer (LIFT), and SERS in an automated system to identify, separate, and characterize single-cell clones for biopharmaceutical production. Post-transfection, Raman spectroscopy revealed distinct IgG-related signals differentiating producing and nonproducing CHO cells, enabling the isolation of individual producers using LIFT for multiwell plate transfer. SERS detected variations in the IgG concentration within the solution.

Schwartz et al. [76] showed promising uses of Raman spectroscopy in upstream bioprocessing, specifically monitoring amino acids and antibody N-glycosylation during high-density perfusion cultures. Probes within bioreactors enabled predictive models for cell density, lactate, ammonium, and amino acids. Graf et al. [77] reported the use of specially designed, single-use in-line flow cells for Raman spectroscopy in a perfusion process’s cell-free harvest stream. Models for glucose and lactate, built using data from five different bioreactor sizes, accurately controlled glucose levels (4 g/L and 1.5 g/L) for days, showing their potential for scale-independent perfusion process monitoring and control. Furthermore, Classen et al. [78] successfully scaled up Raman spectroscopy process analytical technology (PAT) models, initially developed in Ambr^®^ 250 mini bioreactors, to large-scale stirred-tank bioreactors using comparable Raman spectroscopy ports. This technique shows how to quickly and automatically train and validate top-notch Raman models for specific processes in small, high-throughput bioreactors, applicable to larger ones.

Feidl et al. [79] integrated mechanistic modeling with Raman spectroscopy in downstream processing (DSP) to track mAb breakthrough curves during low-titer harvest chromatography, overcoming Raman’s detection limits and noise and achieving adequate control over the capture step. Hauptmann et al. [80] utilized Raman spectroscopy in a study to characterize the proteins in therapeutic mAb IgG1 formulations during freeze–thaw cycles. Protein secondary and tertiary structure in frozen mAb formulations can be tracked via α-helix, β-sheet, random coil, and tertiary structure marker bands. The optimization of freeze–thaw cycles is achievable through methods like cryoprotectant addition or adjusted freezing/thawing rates to prevent protein aggregation. Wei and colleagues [81] applied multiattribute Raman spectroscopy (MARS) to monitor product quality attributes (PQAs) in formulated monoclonal antibody therapeutics. The results show MARS is accurate and reliable for measuring formulation-related PQAs. MARS accurately measured protein concentration, osmolality, pH, and various other concentrations with precision comparable to standard methods, proving highly effective in analyzing protein modifications like fragmentation, aggregation, and oxidation.

Although Raman sensors are common in protein production, their use in cell therapy and viral production is still being researched. Viral-based therapies face numerous challenges, including low production yields, inconsistent batches, and lengthy analytical procedures [82]. Raman spectroscopy offers real-time, extensive process data to overcome these process limitations, thus improving process understanding [83]. Recent studies suggest Raman spectroscopy can measure viral titers [84,85,86]. A rapid, single-use, in-line sensor for this measurement would greatly speed up development and lower manufacturing costs and risks. Real-time vector titer measurement will soon deliver the reliable quality data required for confidence in unit-specific performance. This can shorten the whole process, particularly the later stages.

Despite fewer approved cell-based therapies than protein-based ones, Raman technology sees extensive testing in their processes. Raman spectroscopy effectively distinguishes between various immune cells (B cells, cytotoxic T cells, helper T cells, dendritic cells, and NK cells) [87,88,89]. Despite the similarities in Raman spectra of various immune cells, advanced data analysis [87,88] can distinguish them. Moreover, many studies have demonstrated Raman spectroscopy’s ability to distinguish active and inactive immune cells [87,90,91,92,93]. This is important because most immune cells need to be activated to work. Raman spectroscopy could potentially diagnose immune cell diseases like cancer. Agsalda-Garcia et al. [94] demonstrated the applicability of Raman spectroscopy for diagnosing pediatric non-Hodgkin lymphoma. This method uses a portable fiber optic device to improve Raman spectroscopy technology. This setting enables tissue and cell characterization through a molecular composition analysis, revealing distinct differences or similarities potentially indicative of tumors [94]. For the past ten years, Raman microspectroscopy has been utilized in stem cell therapies to track cell differentiation without labels [95]. New studies show that single-cell differentiation and reprogramming can be identified without labels, using spectral lipid peak biomarkers [96]. Glycogen was used as a biomarker by Hsu et al. to identify neural cell lineage in differentiated human-induced pluripotent stem cells after observing changes in lipid and protein bands. Cell classification was enabled through a developed machine learning method [97]. Kujol et al. distinguished spectral profiles of primary bone marrow mesenchymal stem cells from individual donors. Usual tests of morphology, phenotype, differentiation potential, and markers failed to reveal this information; however, biochemical differences could distinguish between donors [98].

Moreover, the swift approval of mRNA COVID vaccines has fueled the recent rise in mRNA in the gene therapy field. Raman spectroscopy has not been widely adopted yet in this developing field. However, mRNA manufacturing’s adoption of Raman spectroscopy will likely mirror other methods: initial process understanding, then process control. To achieve this, the initial step involves quantifying the components of the in vitro transcription (IVT) reaction. Fed-batch processes can boost productivity [99], and nucleoside triphosphate (NTP) levels can be measured. Visualizing NTP consumption during the IVT reaction enables process control through parameter visualization and boosts productivity by allowing controlled feed additions [84].

## 6. Conclusions

Quantitative polymerase chain reaction (qPCR) is the gold standard for mRNA diagnostic tests. Although qPCR is highly sensitive, it demands a standard lab setting and is quite time-consuming. Besides, the target amplification in qPCR is susceptible to false positives. Molecular diagnostics may revolutionize healthcare in low-resource areas lacking qPCR, enabling faster diagnoses, treatments, and improved prognoses. SERS techniques’ high specificity, sensitivity, and multiplexing capabilities, combined with technological advancements, are well suited for POC molecular diagnostics.

Diverse mRNA applications using SERS spectroscopy employ various strategies to maximize sensitivity and multiplexing. Multiplexed, label-free/direct methods are gaining popularity due to machine learning’s ability to automate data analysis and overcome SERS tag limitations. This approach is still a long way from clinical application. Multiplexed platforms, combining plasmonic nanoparticles, controlled fluid flow, and SERS, achieve the highly sensitive detection of trace mRNA concentrations. Precision oncology may benefit from these SERS-microfluidic platforms, which allow for fast, accurate cancer screening. This technology can significantly improve cancer and other disease screening, leading to better outcomes and earlier diagnoses.

## 7. Future Perspective and Outlook

To ensure the reproducibility and robustness of SERS technology for clinical use, we need rigorously defined protocols and calibration methods. These standards should be developed through collaboration between academic and industry experts, ensuring adaptability to diverse laboratory settings and regulatory needs. Stringent quality control, continuous improvement, and validation will lead to standardized, reliable, and trustworthy SERS applications in clinical diagnostics.

**Figure 5:**
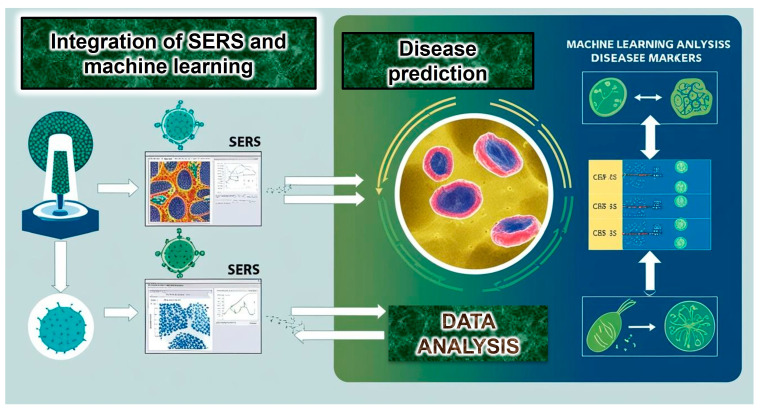
Combining SERS and machine learning allows for highly sensitive, discriminatory prediction of cancer, cardiovascular diseases, and detection of bacteria, viruses, and parasites.

Combining AI/ML with SERS could revolutionize in vivo imaging. These technologies may facilitate analyses of complex in vivo SERS imaging datasets, improving the precise identification of healthy versus diseased tissues. ML algorithms may improve SERS data interpretation, potentially giving clinicians better diagnostic tools and patient outcomes. Future challenges include developing and refining computational models to fully leverage deep learning in real-time clinical applications (Figure 5).

Combining SERS with advanced imaging like OCT and PAI may lead to new multimodal imaging methods offering combined structural, functional, and molecular information. Working together, we can better understand disease at every level, leading to improved diagnoses. Building multimodal platforms using SERS will require creative engineering solutions for smooth data integration and operation, improving their clinical use. Continued research in this field is vital for the translation of these possibilities into practical clinical applications.
